# Surgical management for large vestibular schwannomas: a systematic review, meta-analysis, and consensus statement on behalf of the EANS skull base section

**DOI:** 10.1007/s00701-020-04491-7

**Published:** 2020-07-29

**Authors:** Daniele Starnoni, Lorenzo Giammattei, Giulia Cossu, Michael J. Link, Pierre-Hugues Roche, Ari G. Chacko, Kenji Ohata, Majid Samii, Ashish Suri, Michael Bruneau, Jan F. Cornelius, Luigi Cavallo, Torstein R. Meling, Sebastien Froelich, Marcos Tatagiba, Albert Sufianov, Dimitrios Paraskevopoulos, Idoya Zazpe, Moncef Berhouma, Emmanuel Jouanneau, Jeroen B. Verheul, Constantin Tuleasca, Mercy George, Marc Levivier, Mahmoud Messerer, Roy Thomas Daniel

**Affiliations:** 1 Department of Neurosurgery Service and Gamma Knife Center, University hospital of Lausanne and Faculty of Biology and Medicine, University of Lausanne, Rue du Bugnon 46, CH-1011 Lausanne, Switzerland; 2 Neurosurgical Department, Lariboisière Hospital, Paris, France; 3 Department of Neurosurgery, Mayo Clinic, Rochester, MN USA; 4 Department of Neurosurgery, CHU North Hospital, Aix-Marseille University, Marseille, France; 5 Department of Neurological Sciences, Christian Medical College, Vellore, Tamil Nadu India; 6 Department of Neurosurgery, Osaka City University Graduate School of Medicine, Osaka, Japan; 7 Neurosurgery, International Neuroscience Institute, Hannover, Germany; 8 Department of Neurosurgery, All India Institute of Medical Sciences, New Delhi, India; 9 Department of Neurosurgery, Erasme Hospital, Brussels, Belgium; 10 Department of Neurosurgery, Medical Faculty, Heinrich-Heine-University Düsseldorf, Düsseldorf, Germany; 11 Department of Neurosurgery, University Hospital of Naples Federico II, Naples, NA Italy; 12 Department of Neurosurgery, University Hospital of Geneva, Geneva, Switzerland; 13 Department of Neurosurgery, Eberhard Karls University of Tübingen, Tübingen, Germany; 14 Federal Centre of Neurosurgery, Tyumen, Russian Federation; Department of Neurosurgery, The State Education Institution of Higher Professional Training, The First Sechenov Moscow State Medical University under Ministry of Health, Tyumen, Russian Federation; 15 Department of Neurosurgery, Barts Health NHS Trust, St. Bartholomew’s and The Royal London Hospital, London, UK; 16 Servicio de Neurocirugía, Complejo Hospitalario de Navarra, Pamplona, Spain; 17 Servicio de Cirugía Torácica, Complejo Hospitalario de Navarra, Pamplona, Spain; 18 Department of Neurosurgery, Hopital Neurologique Pierre Wertheimer, Lyon, France; 19 Department of Neurosurgery and Gamma knife Centre, Elisabeth-Tweesteden Hospital, Tilburg, The Netherlands; 20 Signal Processing Laboratory (LTS 5) Ecole Polytechnique Fédérale de Lausanne (EPFL) Lausanne, Lausanne, Switzerland; 21 ENT Service, Centre Hospitalier Universitaire Vaudois (CHUV); Faculty of Biology and Medicine, University of Lausanne, Lausanne, Switzerland

**Keywords:** Large vestibular schwannoma, Combined management, Radiosurgery, Microsurgery, Gross total resection, Subtotal resection

## Abstract

**Background and objective:**

The optimal management of large vestibular schwannomas continues to be debated. We constituted a task force comprising the members of the EANS skull base committee along with international experts to derive recommendations for the management of this problem from a European perspective.

**Material and methods:**

A systematic review of MEDLINE database, in compliance with the PRISMA guidelines, was performed. A subgroup analysis screening all surgical series published within the last 20 years (January 2000 to March 2020) was performed. Weighted summary rates for tumor resection, oncological control, and facial nerve preservation were determined using meta-analysis models. This data along with contemporary practice patterns were discussed within the task force to generate consensual recommendations regarding preoperative evaluations, optimal surgical strategy, and follow-up management.

**Results:**

Tumor classification grades should be systematically used in the perioperative management of patients, with large vestibular schwannomas (VS) defined as > 30 mm in the largest extrameatal diameter. Grading scales for pre- and postoperative hearing (AAO-HNS or GR) and facial nerve function (HB) are to be used for reporting functional outcome. There is a lack of consensus to support the superiority of any surgical strategy with respect to extent of resection and use of adjuvant radiosurgery. Intraoperative neuromonitoring needs to be routinely used to preserve neural function. Recommendations for postoperative clinico-radiological evaluations have been elucidated based on the surgical strategy employed.

**Conclusion:**

The main goal of management of large vestibular schwannomas should focus on maintaining/improving quality of life (QoL), making every attempt at facial/cochlear nerve functional preservation while ensuring optimal oncological control, thereby allowing to meet patient expectations. Despite the fact that this analysis yielded only a few Class B evidences and mostly expert opinions, it will guide practitioners to manage these patients and form the basis for future clinical trials.

## Introduction

Vestibular schwannomas (VS) are histologically benign and typically slow-growing tumors that most commonly arise from the vestibular component of the vestibulocochlear nerve [73]. They represent the most common tumor of the cerebellopontine angle (CPA) and account for about 8% of all intracranial tumors [73]. They usually originate within the internal auditory meatus (IAM) and grow out into the CPA, resulting in an “ice cream cone” shape. These tumors are in most cases sporadic and unilateral with median age at diagnosis increasing gradually from 49.2 years in 1976 to 60 years in 2015 [140]. Approximately 5% of VS are associated with neurofibromatosis type 2 (NF2). In these cases, VS are often bilateral and need to be managed as a separate entity due to their particular characteristics. Patients with NF2 typically present at a younger age and their tumors have a more aggressive or unpredictable growth rate.

Large cystic VS account for 10–20% of all VS and are thought to be characterized by a more rapid growth and worse surgical outcome, mostly related to their larger size and greater adhesion to the surrounding tissues [54], and should be distinguished from solid VS.

VS are commonly associated with biallelic dysfunction of the NF2 gene on chromosome 22 at 22q12.2 which codes for the tumor suppressor protein schwannomin or merlin [24, 45].

In the early twentieth century, Harvey Cushing significantly refined the surgical technique introducing the wide bilateral suboccipital craniectomy with subcapsular subtotal resection instead of total finger enucleation, thus reducing mortality to < 20% [103]. Subsequently, Cushing’s student, Walter Dandy advocated a unilateral approach and gross total removal (GTR) to reduce recurrence [103].

Despite the improvement of surgical technique, mortality remained high in the hands of less experienced surgeons. Even extremely accomplished surgeons (Olivecrona 1891–1980) [50] experienced significant morbidity when operating large tumors with a mortality rate up to 20% and 5 times that of a small tumor [122]. Despite further technical evolution, the correlation between size and outcome still exists and characterizes the complexity of the surgical management of large VS. The introduction of the operating microscope, arguably one of the most important technological advancements has increased the capability of GTR and reduced the mortality and morbidity [103]. The introduction of intraoperative neuromonitoring (IOM) techniques has further improved preservation of neurovascular structures during tumor excision.

In parallel, the development of noninvasive, focused radiation therapy techniques introduced in the 1950s by Lars Leksell [95] would evolve into modern stereotactic radiosurgery (SRS) which currently represents a valuable alternative treatment for small-medium sized lesions, with a high rate of tumor control and functional nerve preservation [111, 112, 133, 139].

However, despite these advances, treatment and management of large VS remains challenging and GTR is still associated with a high risk to facial and cochlear nerve function [2, 77, 144].

## Methods

This work represents the consensually derived opinion and recommendations of the EANS skull base section board with the valuable participation of invited renowned experts in this field after a systematic review and meta-analysis of studies in literature, followed by formal discussions within the group.

Following PRISMA guidelines and recommendations, we conducted a systematic search using the MEDLINE database without backward date limit. The following medical subject headings (MeSH) and free text terms were combined: “acoustic neuroma” OR “vestibular schwannoma” AND “Surgery” OR “operative surgical procedures” OR “Outcome” OR “radiology” OR “epidemiology” OR “screening” OR “Radiation” OR “Radiosurgery” OR “Recurrence” OR “Quality of life” OR “cranial nerve monitoring.” No language restrictions were applied. The “related articles” function was used to obtain any relevant reports. We manually reviewed the reference lists of identified studies for further inclusions.

After having defined “large VS” as tumors larger than 30 mm and giant tumors > 40 mm (“surgical classification” section), we performed “a posteriori” subgroup analysis screening of all surgical series published within the last 20 years (January 2000 to March 2020) for a qualitative synthesis. Studies were eligible if they met the following criteria: (1) included a group of at least 10 patients, (2) included patients with large and/or giant VSs (as previously defined), and (3) microsurgical resection which represented the first-line treatment. Large series encompassing all sizes of VS without a subgroup analysis were excluded. We also excluded studies that included patients with neurofibromatosis type 2 and in which the reported outcome data after GTR were not distinguishable from cases undergoing a near-total or subtotal resection (STR). When duplicate studies were published with accumulating numbers of patients or increased duration of follow-up, only the one reporting the entire necessary outcomes was included. Eligibility was independently assessed by two authors (D.S. and L.G.), and differences were resolved with the help of a third author (R.T.D.). Weighted summary rates were determined using meta-analysis models. Pooled estimates using meta-analytical techniques were obtained for the rate of total resection, oncological control, and facial nerve preservation after gross total resection. We had earlier published a meta-analysis based on the pooled results of patient series treated with subtotal resection and stereotactic radiosurgery [158]. The results of the two meta-analysis and the systematic review of literature were discussed within the task force to generate recommendations to arrive at a consensus. Quality of evidence was assessed using the Grading of Recommendations, Assessment, Development and Evaluation (GRADE) Working Group system [6, 53]. If randomized blinded trials or prospective matched-pair cohort studies were identified, the recommendations were Level A or B. For controlled nonrandomized trials or uncontrolled studies, the recommendations were Level C or “expert opinion,” respectively.

## Growth pattern and measurement of tumor size

The reported growth rate of isolated untreated VSs varies widely from 15 to 85% [185], depending on the population and length of the observation period. Indeed, most observational studies often include a subpopulation of patients with small tumors or patients not eligible for surgery. To date, the largest data comes from Denmark, where a national database maintained since 1975 includes more than 2500 patients [157]. This report estimates that approximately 29% of the extrameatal tumors increased in size within the first 5 years after diagnosis with higher mean annual growth during the first year (62% of tumors). In contrast, other series probably overestimate the rate of tumor growth as patients are referred only after growth has been detected [93].

Assessing tumor growth rate may also depend on the chosen criteria for the determination of growth (largest diameter vs. volume, number of millimeters or cubic centimeters). In the Danish database, tumor growth was defined as an increase of at least 3 mm in the largest extrameatal diameter, but the adequacy of this parameter has been widely questioned since a tumor may grow along other directions and may be missed with traditional linear measurements [93, 150]. With a mean follow-up of 4.1 years, Lees et al. [93] reported that approximately 51.2% of the extrameatal tumors showed progression (defined as ≥ 2 mm increase in tumor diameter) at a median linear diameter growth rate of 1.49 mm per year. When assessing the volumetric growth (defined as ≥ 20% increase in volume, based on literature evidence which showed that data error as high as 20% should be considered when assessing volumetric changes[126]), they found that 67.4% of the extrameatal tumor showed progression at a rate of 32.9% change in volume per year in accordance with previous reports [150, 173, 185]. Schnurmann et al. [150] assessed tumor growth rates using volumetric measurements in 212 patients and found that 66% of the tumors demonstrated growth over an average interval of 25 months with a volumetric growth rate of 33.5% per year. In this series only 8% of the extracanalicular tumors were larger than 3 cm. Since small-medium sized VSs represent the vast majority of the tumors analyzed in these studies, the applied tumor growth cutoffs may not be adequate for large VS for whom even a minor linear growth can result in a substantial increase in volume, considering that a 6% increase along one axis corresponds to a 20% volumetric growth in a sphere [150]. To date there is no consensus on the quantitative definition of growth in terms of linear or volumetric cutoff.

The 1995 American Academy of Otolaryngology-Head and Neck Surgery Foundation consensus guidelines recommended the use of two linear measurements, the diameter of the tumor parallel to the petrous ridge and the maximum diameter of the tumor in the orientation perpendicular to the first one and then use the square root of the product of these two measurements as the tumor size; these measurements may provide a good indication of the position of the cranial nerves and the degree of brainstem compression [124]. In an effort to standardize the reporting of tumor measurements, the 2003 consensus meeting [79] agreed that tumor size should be based on linear planimetric measurements of the largest extrameatal diameter on a post-contrast axial magnetic resonance image (MRI).

*The literature supports the use of tumor size for reporting results. For large* VS *the largest extrameatal diameter of the tumor and its volume should be described. The literature does not provide enough evidence to support the use of planimetric* vs. *volumetric measurement to assess tumor size and growth. (Expert opinion)*

### Surgical classification

In order to improve the understanding of VS and to compare the results of management, numerous efforts have been made to classify and characterize these lesions according to their size and surgical anatomy. Since the size of the intrameatal part, no matter how large, does not affect the management of the disease, most classifications have concentrated on characterizing the extrameatal portion and its anatomical relationships.

Early grading systems proposed by House [63] and Sterkers [159] were based solely upon measurements of the extrameatal maximum diameter and categorized tumors by relative size in qualitative categories (e.g., mild, large, huge or Grades IV and V). Large VSs were defined as a tumor measuring > 30 mm. A classification based purely on planimetric measurements does not take into account other anatomical factors that influence surgical management such as brainstem compression and/or deformation of the 4th ventricle.

The Koos classification [83] combined extrameatal tumor size and the anatomical description defining a tumor up to 3 cm as Grade III which occupies the CPA but does not compress the brainstem, and as Grade IV (large tumors) a tumor which compresses and displaces the brainstem and measures more than 3 cm. The Hannover classification [144] also categorizes VS according to the relationship with the brainstem such that T4 represents those tumors in contact with the brainstem and causing a mass effect with further subclassification into T4a and b based on severity of brainstem compression and fourth ventricle deformation. Despite a high intra- and inter-rater reliability [37], it is difficult to translate these anatomical classifications into a geometric classification and vice versa in order to compare data from different series. The 2003 consensus meeting on systems for reporting results in vestibular schwannoma [79] agreed that tumor size measurements on the post-contrast axial MRI should use linear planimetry with 10-mm increments on the largest extrameatal diameter. According to these criteria, larger tumors were classified as large (31–40 mm) and giant > 40 mm.

*The literature supports the use of anatomical classification when reporting the results of* VS *surgery as they enable comparison between series. In order to standardize tumor classification we recommend using the largest extrameatal tumor diameter on the axial MRI and grouped in 10 mm increment intervals. According to this classification, large* VS *are defined as tumors larger than 30 mm and giant tumors > 40 mm. (Expert opinion)*

### Clinical screening and evaluation

With rising healthcare costs and resource utilization, an optimal screening method for VS is still a matter of debate due to the lack of sensitive and specific symptom-based tests. Presenting symptoms may be insidious at onset, progressing from early asymmetric sensorineural hearing loss (ASNHL) and/or vestibular dysfunction to symptoms of brainstem compression and eventually hydrocephalus.

ASNHL, often misinterpreted as age-related hearing loss, is generally insidious and is the initial complaint in three quarters of patients. Despite the etiological heterogeneity attributed to ASNHL, audiometric findings of ASNHL of ≥ 10 dB at two or more contiguous frequencies or ≥ 15 dB at any single frequency suggest a diagnosis of a VS with an average 93% sensitivity and a low specificity (< 70%) [49, 143]. In order to increase the specificity for clinical diagnosis, several authors have analyzed a variety of presenting symptoms in VSs [102]. Continuous, ipsilateral, asymmetric high-pitched tinnitus is seen in 70% of patients with VS and ASNHL. Despite this, only a minority (less than 1%) present with tinnitus as initial presenting symptom, suggesting that this symptom was more related to ASNHL rather than the tumor [102]. Asymmetric tinnitus alone is a nonspecific symptom and an unreliable indicator of the presence of a VS.

BAERs do not play a major role in the diagnosis of VSs; however, a pooled meta-analysis showed that they retain a 86% sensitivity (up to 96% for larger tumors) and 82% specificity and are especially cost-effective for patients at low-risk for VSs based on clinical and audiological findings [82]. Patients with large VSs may additionally present with signs and symptoms of other cranial nerve and brainstem dysfunction in up to 80% of cases [144, 146]. Gait instability due to cerebellar or vestibular pathway impairment, long tract signs, and symptoms of intracranial hypertension have been associated in more than 30–50% of patients [144, 146]. Combinations of presenting signs and symptoms (ASNHL, pulsatile asymmetric tinnitus, dizziness, and localizing posterior fossa signs/symptoms) have a specificity for VSs of 99% [61]. Localizing posterior fossa or CPA signs/symptoms with or without audiovestibular symptoms urge further investigation with MRI.

#### Preoperative assessment of hearing and facial functions

Classically, the two mainstream classification systems for audiogram findings are the Gardner-Robertson (GR) and American Academy of Otolaryngology-Head and Neck Surgery (AAO-HNS) classifications [46, 123]. Consensus is lacking on what characterizes useful or serviceable hearing. AAO-HNS Class A and Class B are considered to be “useful” or “serviceable” hearing in the AAO-HNS system, and they are equivalent to GR Grades I and II with a pure tone average (PTA) ≤ 50 dB and/or a speech discrimination (SD) score > 50% at 50 dB. Nevertheless, many authors have criticized these PTA and SD thresholds that may overestimate hearing function, questioning their relevance in socially useful hearing [79]. Therefore, the 2003 consensus meeting proposed a new classification in 6 classes (A–F) in which serviceable hearing (Class A–B) was defined as a ≤ 30-dB PTA and a > 70% maximum speech discrimination score which correspond to GR Grade I and AAO-HNS Class A only. However, the lack of homogeneity in reporting audiological data makes its interpretation and comparison cumbersome.

The House-Brackmann (HB) grading scale is the most used classification for facial function, enabling comparison between surgical series with a relative small inter-observer variability [65]. Grades I and II (normal and mild dysfunction) are accepted worldwide as a “functional” status and regarded as a satisfactory treatment outcome and Grades IV and V as “nonfunctional” and unsatisfactory. Grade III (moderate dysfunction) remains controversial as most series classify it as “functional/satisfactory” whereas these patients are at a higher risk of developing keratitis sometimes needing a tarsorrhaphy or upper eyelid gold weight placement [79].

*The literature supports an initial screening evaluation with audiometry for all patients with symptoms of hearing impairment and accompanying symptoms such as vestibular dysfunction and/or non-localizing clinical signs. An audiometric ASNHL pattern of hearing loss with ≥ 10 dB at 2 or more contiguous frequencies or ≥ 15 dB at any single frequency should prompt a screening MRI. Regardless of the presence of audiovestibular dysfunction, the literature suggests that all patients with clinical signs and symptoms of brain stem dysfunction and/or hydrocephalus undergo MRI screening. (Level C)*

*The literature supports the use of grading scales for pre- and postoperative hearing and facial nerve function when reporting outcomes after treatment to allow meaningful comparison between different treatment or surgical approaches and across different series. There is lack of consensus regarding the audiometry thresholds to define socially serviceable hearing. Due to their worldwide application and their direct overlap for serviceable status, literature supports the use of the AAO-HNS or GR classification for reporting hearing function. Due to its worldwide application and small inter-observer variability the literature supports the use of the HB grading system for facial nerve function. Consensus is lacking regarding the definition of functional/satisfactory status. (Expert opinion)*

### Preoperative imaging

Computed tomography (CT) provides essential anatomical information of the petrous bone such as pneumatization, presence of high jugular bulb or large emissary veins, and can be useful in surgical planning. MRI is superior in evaluating CPA pathology [163] with standard T2, pre- and post-gadolinium T1 and diffusion weighted imaging (DWI) providing high sensitivity (96–100%) and specificity (90–93%) for detecting VS [56, 155]. The development of 3D imaging techniques has allowed further improvement in the sensitivity and specificity of diagnostic techniques and currently high-resolution T2 constructive interference in steady state (CISS) and post-contrast T1 magnetization prepared rapid acquisition gradient echo (MPRAGE) sequences allow an excellent identification of neurovascular structures in the CPA, providing valuable preoperative information concerning internal tumor architecture, its boundaries, and anatomical relationships in the axial, coronal, and sagittal planes [60, 125].

Early identification of the course of the facial nerve (FN) at surgery should be facilitated if the position and course of the nerve can be demonstrated on the preoperative image. High-quality T2 imaging techniques highlighting tissue-fluid interface, such as CISS and fast imaging employing steady-state acquisition (FIESTA) MRI, delineate the FN position with high sensitivity and reliability in small to moderate size tumors; however, it becomes more challenging in the case of large VSs due to nerve thinning and anatomical landmark distortion [115, 148].

These drawbacks have been partly overcome, even in large VSs, by improvements in diffusion tensor imaging-fiber tracking (DTI-FT) “tractography” [74, 75] that is able to delineate cranial nerves “displaced” by the tumor in 80 to 100% of the cases studied [74] [149]. At present, this technique is being progressively utilized for academic and clinical purposes in a few centers [74, 128] and requires validation through further clinical experience.

*The literature supports the use of MRI and CT scan for initial preoperative imaging of a* VS. *MRI represents the gold standard and the literature supports the use of high-resolution T2 and gadolinium-enhanced T1-weighted MRI in axial, sagittal and coronal planes for detection of* VS. *High quality T2-imaging techniques (CISS, FIESTA) and tractography-reconstruction imaging (DTI-FT) may be used to increase visualization of FN course within CPA. However, the impact of these imaging techniques on routine clinical application and on functional outcome needs to be validated by further clinical experience. (Level C)*

### Management goal and strategy

#### Total resection

The treatment and management of VS has dramatically evolved over the last decades; currently, the goal of the management should be focused on tumor control and on maintaining or improving the quality of life (QoL) of the patient with low morbidity and better neurological function preservation.

Although SRS for small-medium sized lesions represents a valuable alternative treatment [111, 112, 133, 139], it is less frequently employed for large VSs because of the need for surgical decompression and the risk of clinical deterioration during transient tumor expansion after SRS [158].

Microsurgical GTR is associated with low tumor recurrence rates, reported to be between 0% and 9.1% (Table 1), and a pooled overall tumor control rate of 99.8% (95% CI 99.5–100%) (Fig. 2), compared with a rate of tumor remnant progression between 30 and 80% after a subtotal resection (STR) [12, 44, 116].

**Table 1:** Results of patient series treated with gross total resection for large vestibular schwannomas

Author (publication year)	Number of patients	Surgical approach TL/TO RL RS			GTR rate	Mean follow-up (months)	FN preservation % (HB I–II) after GTR	CN preservation (%) after GTR	Tumor control (%) after GTR
Wu et al. 2000	40	100%	–	–	97.5%	3 (6–10)	65%	NR	100%
Jung et al. 2000	30	–	–	100%	73.3%	NR	36.4%	NR	100%
Sluyter et al. 2001	99	100%	–	–	91.7%	(8–24)	50%	0	NR
Mamikoglu et al. 2002	81	100%	–	–	95.1%	> 12	45%	0	100%
Lee et al. 2002	36	–	–	100%	30.6%	24	66.7%	0%	100%
Yamakami et al. 2004	50	–	–	100%	86%	58	84%	2%	100%
Roland et al. 2004	56	82%	–	18%	73.2%	29	84%	NR	100%
Darrouzet et al. 2004	152	76.9%	17.8%	5.3%	98.7%	70	NR	NR	NR
Sanna et al. 2004	175	100%	–	–	85.1%	> 12	29.6%	0%	100%
Gerganov et al. 2005	18	–	–	100%	61.1%	12	39%	0%	NR
Darwish et al. 2005	35	–	–	100%	NR	NR	22%	0%	93.8%
Raftopoulos et al. 2005	16	6.3%	–	93.7%	68.8%	55	100%	50%	90.9%
Anderson et al. 2005	71	69%	–	31%	95.8%	6	73.2%	NR	100%
Zhang X. et al. 2005	105	–	–	100%	86.7%	NR	56.3%	0%	100%
Jain et al. 2005	145	–	–	100%	97.9%	(6 w–11 y)	30.4%	NR	NR
Samii et al. 2006	92	–	–	100%	95.7%	24	52%	28.6%	98.9%
Liu et al. 2007	19	–	–	100%	63.2%	3–10	63.2%	NR	NR
Cardoso et al. 2007	166	–	–	100%	98.8%	NR	NR	0%	98.8%
Strauss et al. 2008	10	–	–	100%	70%	35	60%	10%	100%
Chen et al. 2009	39	–	–	100%	NR	16	69.2%	NR	NR
Wanibuchi et al. 2009	16	–	–	100%	NR	(24–108)	NR	56.2%	NR
Charpiot et al. 2010	123	100%	–	–	96.7%	> 12	68.5%	0%	100%
Zhao et al. 2010	89	–	–	100%	42.7%	NR	54%	NR	100%
Talfer et al. 2010	51	100%	–	–	NR	45	49%	NR	NR
Bloch et al. 2011	100	NR	NR	NR	NR	37	44%	NR	NR
Di Maio et al. 2011	47	–	–	100%	87.2%	NR	93.6%	21.4%	100%
Raslan et al. 2012	47	59.6%	–	40.4%	89.4%	36	70.2%	0%	100%
Silva et al. 2012	29	–	–	100%	100%	39	44.8%	0%	100%
Zhang Zh. et al. 2012	115	100%	–	–	89.6%	(12–60)	35.7%	0%	98%
Nonaka et al. 2013	62	NR	NR	NR	45.2%	> 24	66.9%	NR	NR
Porter et al. 2013	153	100%	–	–	35.9%	> 12	78.2%	0%	NR
Lim et al. 2013	27	–	–	100%	NR	40.1	74.1%	NR	100%
Daming et al. 2014	37	–	–	100%	94.6%	> 12	81.1%	5.7%	NR
Moffat et al. 2014	145	94.2%	–	5.8%	NR	> 24	44.14%	0%	NR
Liu S. et al. 2015	106	–	–	100%	82.1%	24	78.3%	NR	100%
Turel et al. 2016	179	–	–	100%	86%	18.1	35.2%	0%	NR
Zhang S. et al. 2016	218	–	–	100%	26.6%	39.7	58.6%	9.6%	96.6%
Zhang Z. et al. 2016	186	100%	–	–	97.8%	70.8	79.9%	0%	NR
Huang et al.2017	657	–	–	100%	84.6%	59.6	32.9%	7.14%	100%
Boublata et al. 2017	151	–	–	100%	83.4%	28	82%	NR	NR
Hoshide et al. 2018	45	–	–	100%	64.4%	49	84.4%	37.5%	100%
Breun et al. 2019	320	–	–	100%	61.3%	NR	58.5%	12%	100%
Troude et al. 2019	169	36%	–	64%	11%	62	NR	NR	NR

*TL* translabyrinthine, *TO* transotic, *RL* retrolabyrinthine, *RS* retrosigmoid, (GTR) gross total resection, *FN* facial nerve, *CN* cochlear nerve, *NR* not reported

**Fig. 1:**
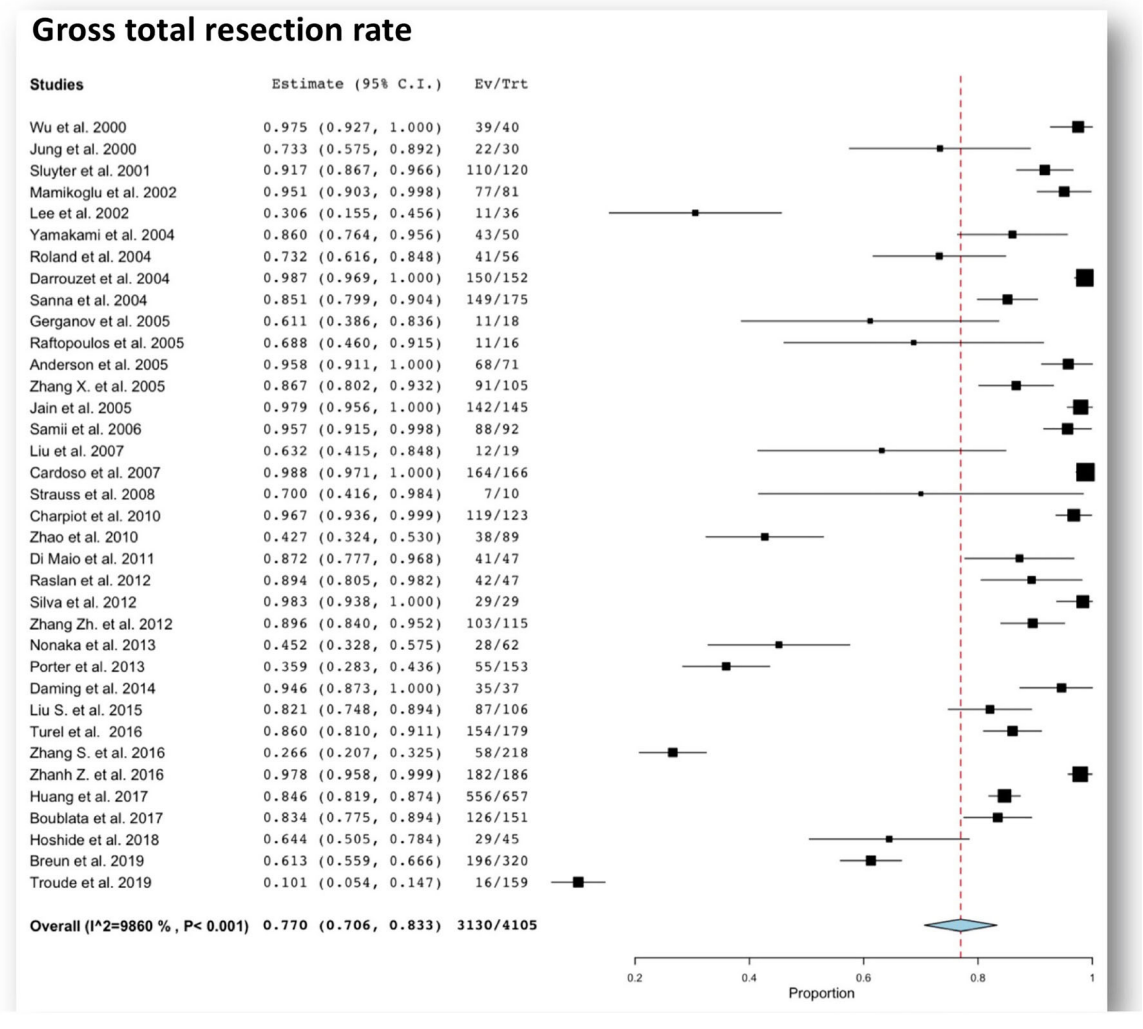
Forest plot graph showing pooled rates of gross total resection rate after total resection for large vestibular schwannomas. The meta-analyzed measure is plotted as a diamond. The summary measure (center line of diamond) shows a gross total resection rate of 77%. The associated confidence intervals correspond to the lateral tips of the diamond

**Fig. 2:**
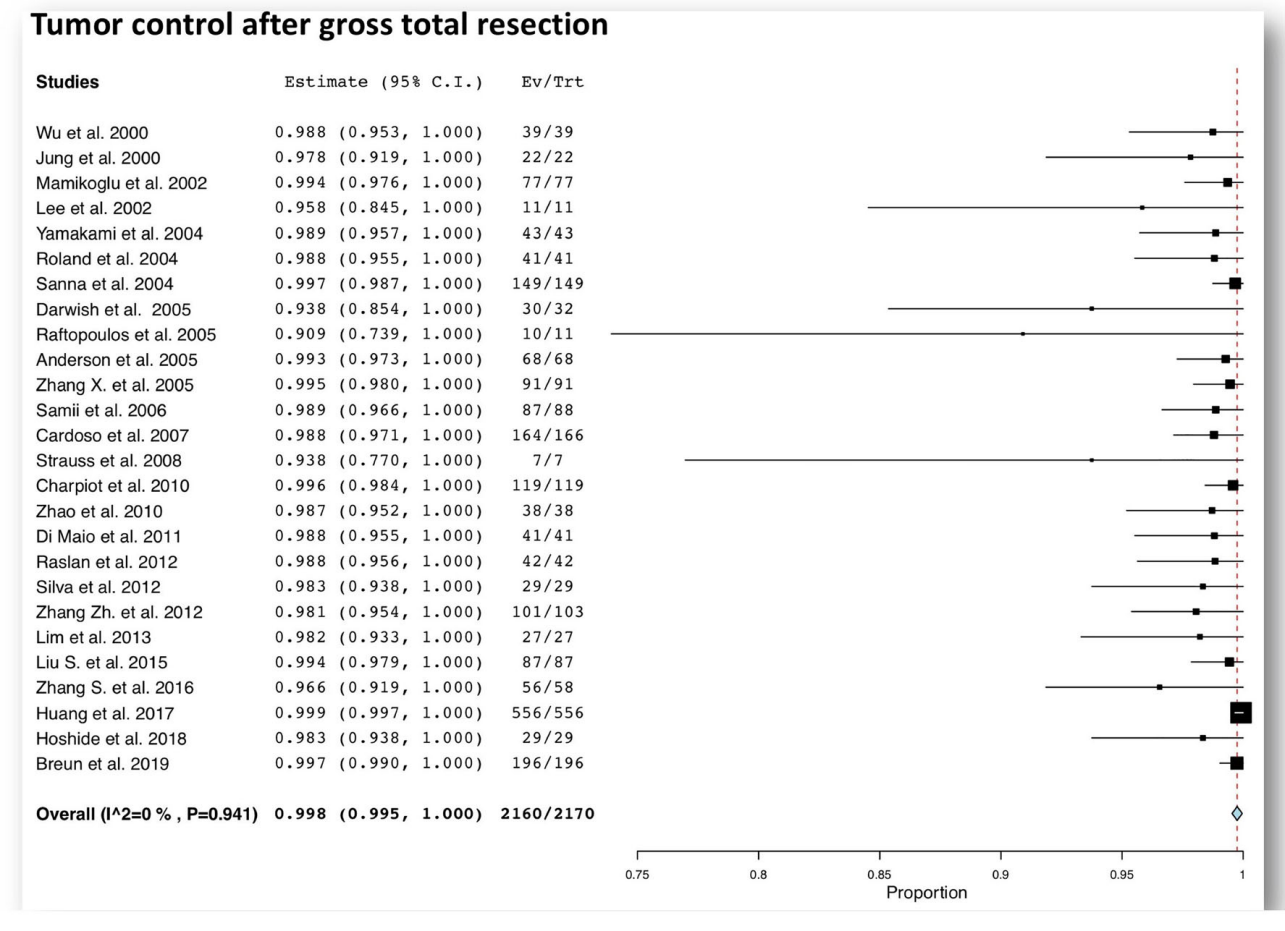
Forest plot graph showing pooled rates of tumor control after total resection for large vestibular schwannomas. The meta-analyzed measure is plotted as a diamond. The summary measure (center line of diamond) shows a oncological tumor control rate of 99.8%. The associated confidence intervals correspond to the lateral tips of the diamond

Beyond the excellent oncological control, completely removing the tumor could also have a significant impact on the quality of life and represents a psychological advantage to the patient [98]. Despite the best of surgical techniques and electrophysiology equipment, surgical outcomes are still bound by tumor characteristics, such as size, and depend predominantly on the individual surgeon’s experience and skill. In experienced hands, GTR can be achieved in more than 90 up to 100% of cases (Table 1). The pooled overall GTR rate was 77% (95% CI 70.6–83.3%) in large series of large VS (Fig. 1) [2, 13, 14, 17, 19, 25, 26, 30, 33–35, 47, 64, 66, 76, 77, 88, 97, 99, 100, 104, 109, 119, 134, 137, 138, 142, 145, 147, 152, 156, 160, 161, 166, 169, 177, 180, 181, 186–190]. Notably, high-volume hospitals and surgeon caseload have been associated with decreased mortality, decreased postoperative complications and readmission rate, and better oncological and functional outcome [7, 8] (Fig. 2).

This volume-outcome effect has an even greater impact on functional nerve preservation. A learning curve has been well established and each annual increase in case-load of at least 10 patients has been associated with a significant decrease in the complication rate and better functional outcome [7].

Nevertheless, large tumors are more likely to result in facial paralysis and hearing loss when compared with small tumors [178].

**Fig. 3:**
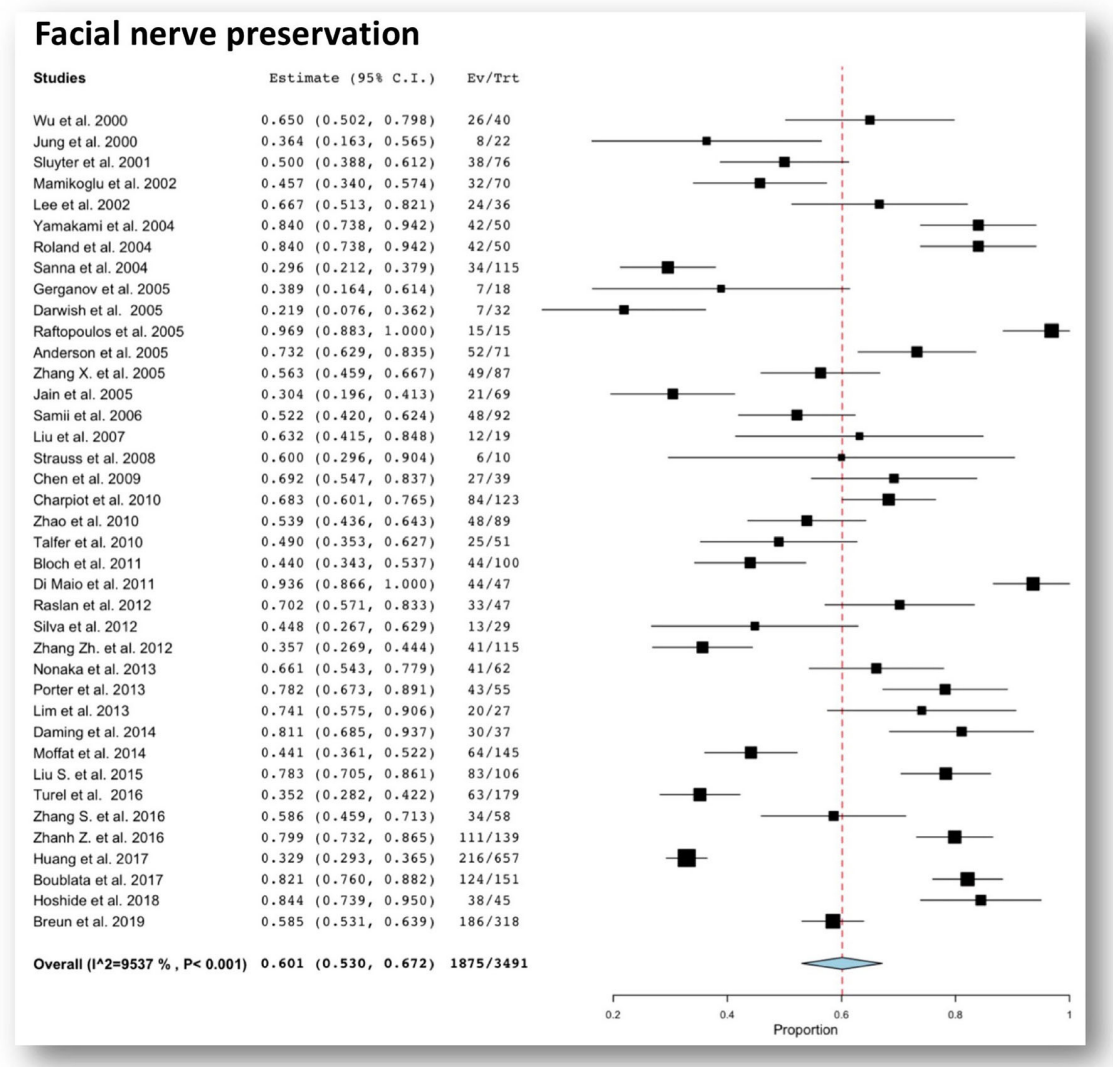
Forest plot graph showing pooled rates of functional facial nerve preservation after total resection for large vestibular schwannomas. The meta-analyzed measure is plotted as a diamond. The summary measure (center line of diamond) shows a facial nerve functional (HB 1-2) rate of 60.1%. The associated confidence intervals correspond to the lateral tips of the diamond

FN function preservation rates vary according to surgeon experience; in a large series of large VS operated with more than 90% rate of GTR, when strict criteria of FN function preservation are applied (HB ≤ 2), a satisfactory outcome is achieved in 30 to 84% of cases (Table 1). Overall the pooled rate of facial nerve preservation in a series of large VS was 60.1% (95% CI 53–67.2%) (Fig. 3).

Despite numerous authors asserting that anatomical and functional cochlear nerve preservation cannot be accomplished in case of GTR for large VS [5, 184], the philosophy of microsurgery has changed to include attempts at preservation of cochlear nerve function (in patients with serviceable hearing). Samii [145] comparing the results of his two large series reported an overall increase in the rate of anatomical cochlear nerve preservation from 68 to 75.8%. However, the rate of functional preservation decreases with increasing tumor size, and hearing preservation rates after GTR in large VS remained low at 28.6%.

The translabyrinthine approach is frequently employed in large VS, assuming that the likelihood of preserving hearing is poor. However, when a hearing-preservation technique is used, the rate of cochlear nerve functional preservation ranges from 2 to 56.2%, as reported by several series on large VS (Table 1).

#### Less than total resection

STR is associated with good facial and cochlear nerve functional preservation rates of 90% and 80% respectively; however, the recurrence rate has been shown to be intimately related to the residual tumor volume with a risk of tumor progression of > 50% [172]. The definition of a near-total resection (NTR) vs. STR is not standardized and varies between different series; some authors define a NTR as more than 95% excision [55], others as a residual tumor of 25 mm^2^ or of a 2-mm thick pad [12] and in most cases the residual volume is described as a percentage of initial volume [52]. No standard evidence-based definition of a NTR vs. STR has been established and consequently no conclusion can be drawn [52].

Over the last decade, the increased functional outcome expectations in patients harboring large VS have led to a progressive shift of focus in the expectations of vestibular schwannoma surgery. Several series have now reported their results where the functional nerve preservation has assumed as much of an importance as oncological control [31]. This has led many centers to perform STR and subsequent SRS, either upfront or because of growth of tumor remnants at follow-up serial imaging (Table 2) [31, 43, 55, 70, 71, 110, 127, 129, 136, 174, 183]. A few series have, to date, published their results with this approach (Table 2), reporting HB Grades I–II in 96.1% of patients (95% CI 93.7–98.5%), while serviceable hearing was maintained in 59.9% (95% CI 36.5–83.2%) [158]. A recent meta-analysis [158] of this combined approach showed a progression-free survival of 93.9% at a mean follow-up of 36.9 months. This strategy was recently validated by the European Association of Neuro-Oncology as a valid option in large VS [51].

**Table 2:** Studies of patients treated with subtotal resection and stereotactic radiosurgery

Author (publication year)	Number of patients	Mean follow-up (months)	% Facial nerve preservation (HB I–II)	Cochlear nerve preservation (%)	Tumor control (%)
Iwai et al. (2003)	14	32	85.7	NA	79
Park et al. (2006)	8	68.8	NA	NA	100
Yang et al. (2008)	61	53.7	95	NA	93.5
Fuentes et al. (2008)	8	46	87.5	NA	100
Van de Langenberg et al. (2011)	50	33.8	94	25 (1/4)	90
Haque et al. (2011)	151	72	97	–	87
Pan et al. (2012)	18	57	89	100 (11/11)	100
Iwai et al. (2015)	40	66	95	42.9 (6/14)	90
Radwan et al. (2015)	22	28	87	NA	100
Monfared et al. (2016)	73	38	81	–	79
Daniel et al. (2017)	32	29	100	76.9 (10/13)	91.6

*NA* not assessed

Nevertheless, these data are based mostly on retrospective series with low or very-low quality of evidence [158] and none of them offer a direct comparison with a group of GTR and have limited follow-up intervals of about 3 years. Therefore, further prospective studies are necessary to provide a stronger support to this strategy.

#### Planned subtotal vs. unplanned subtotal resection

During the last decade, some centers coined the term “planned subtotal resection” that implies focusing on mass effect decompression and on rendering the residual tumor volume suitable for SRS [158] (Table 2). The goal of this strategy is to obtain a uniform thickness of the residual tumor capsule covering the nerves and no dissection is attempted between the plane of the nerve and the tumor capsule, thereby maximizing the chance of preserving function [31]. Studies reporting on this technique showed a tumor control rate similar to that of the series on GTR with higher rates of FN function preservation [31, 158]. A second strategy utilizes an unplanned STR where the surgeon makes an intraoperative decision to halt the resection when it is felt that further tumor removal will jeopardize the FN function [16, 43, 129, 183]. Interpreting the outcome of this second strategy is difficult, as there are many confounding factors such as the experience of the surgeon in modifying his/her strategy according to the specific case. Additionally, the outcome may be quite different if the decision to stop the resection is taken prior to any dissection of the capsule from the nerve versus halting the surgery after several dissection attempts. These variables explain the wider range of reported outcome in terms of FN preservation between 40 and 95% [16, 43, 129, 183]. No direct comparison between these two strategies is available in literature; hence, no consensus can be reached. None of the published studies have their own internal control and are all retrospective series with low or very-low quality of evidence.

#### Microsurgical approaches

Historically the main microsurgical approaches for VSs resection and FN preservation were either the middle fossa (MF) or the retrosigmoid (RS) approach when serviceable hearing is present, and a RS or translabyrinthine (TL) approach when serviceable hearing is not present. Regarding hearing preservation, class III evidence studies failed to show superiority of one surgical approach over another mainly due to selection biases when tumor size was not adjusted for [62, 135]. The MF approach has been used for small intrameatal tumors, whereas larger medially located lesions are generally approached through a RS craniotomy. In the case of large tumors, FN preservation up to 98% and hearing preservation ranging from 10 to 68% are reported with the RS approach [62, 119, 182]. It may be noted that results from these series are confounded as they are not adjusted for size and preoperative functional status. In the absence of preoperative serviceable hearing, both the RS and the TL approaches have been used with the intent of a GTR [33, 36, 39, 104, 108, 109, 187]. Results from retrospective or nonrandomized prospective series are discordant regarding functional preservation and significant variability related to tumor size and surgeon’s preferences do not allow for definite conclusions. Once again, class III evidence data failed to show superiority of one approach over the other [33]. Analysis of pooled data from large VS resection through a RS showed a GTR rate of 79.1% (95% CI, 64.2–90.8%) with a good functional FN outcome (HB Grades I and II) in 62.9% (95% CI, 50.0–74.9%) of cases [191].

Several centers advocate staged resection for large VSs to improve the resection quality and functional nerve outcome; however, it is not clear whether this strategy translates to improved facial nerve outcomes with fewer complications. Raslan et al. [138] compared the results of a cohort of 28 patients undergoing staged resection with those of a similar cohort of patients who underwent a single-stage resection. After a first-stage retrosigmoid approach, the decision to stage the resection was taken intraoperatively in case of cerebellar or brainstem edema, tumor adherence to the brainstem of the facial nerve, or in case of a thin, poorly-visualized facial nerve. In these cases a second-stage translabyrinthine approach was performed at a later date. The authors reported that a staged resection was associated with a higher rate of GTR (96.4% vs. 79%, *p* < 0.01) and better facial nerve outcome (HB I–II, 82% vs. 53%; *p* < 0.01), without added neurological morbidity. Porter et al. [134], similarly, reported the results of a group of 75 patients undergoing staged resection through a first-stage retrosigmoid approach and a second-stage translabyrinthine approach. Compared with a group of patients undergoing one-stage surgery, the authors reported similar rates of GTR and facial nerve outcome (HB I–II, 81% vs. 76%). The authors observed that after the first stage, the nerve became more robust and resistant to additional manipulation allowing a complete removal which was initially halted during the first surgery. Patni et al. [130] reported on 34 patients for large VS in which staging was planned preoperatively. All patients had total or near-total resection with no recurrences, and 94% had HB I–II facial nerve function at the latest follow-up. These results are from retrospective studies, and therefore have inherent limitations and biases due to the arbitrary selection of surgical strategy by the surgeon on a case-by-case context. There is also a concern whether additional surgeries are related to more complications, which has not as yet been documented in literature.

*According to recent literature, the goals of* VS *management should be primarily focused on maintaining or improving QoL making every attempt at neurological function preservation with an acceptable oncological control. (Expert opinion)*

*Patients with large VSs should be counseled about the risk of functional facial and cochlear nerve impairment associated with surgical treatment and the specific strategy should be tailored according to the patient’s expectations. In the European context, patient expectations should be taken into careful consideration while deciding on the surgical strategy with a view to impart as much of an importance to functional nerve preservation as to tumor excision. (Expert opinion)*

*There is insufficient evidence in literature to support the superiority of one surgical strategy over another for resection of large VSs and functional outcome preservation. (Level C)*

### Intraoperative cranial nerve monitoring

#### Facial nerve mapping and monitoring

Preservation of the FN has of late become the primary benchmark reported by all recently published series. Advances in microsurgical instrumentation and techniques along with the all-important contribution of intraoperative monitoring of the cranial nerve function have led to a significant improvement of functional outcome and patients’ QoL [96, 153]. The role of FN monitoring has shifted, over the years, from a simple identification and intraoperative mapping of the nerve to an intraoperative prognostic indicator of functional preservation and long-term outcome [96, 153]. At present, FN IOM represents the standard of care and is considered an indispensable tool for the surgical management of VSs. However there exists a dearth of controlled data comparing monitored and unmonitored surgeries. In fact, most of the available evidence goes back to the historical series [86, 96, 153], which prospectively analyzed the functional outcome after VS resections in the pre- and post-IOM eras, showing the clear benefit of FN monitoring in terms of function preservation.

Intraoperative FN monitoring, using electrically evoked testing with free-running electromyography (EMG) and compound muscle action potential (CMAP), is commonly used during VS surgery. Electroprognostic factors such as post-resection nerve stimulation thresholds at the level of the nerve root entry zone brainstem, response amplitudes, and tonic/train activity on continuous EMG monitoring have been proved to predict a good functional outcome after tumor resection [96, 153, 176]. Nevertheless, the absence of electrophysiological responses or spontaneous tonic/train activity, in case of an anatomically intact facial nerve, is not an ineluctable indicator of permanent FN paralysis [21]. Facial EMG recordings following cortical stimulation of the facial motor area (so-called facial MEP) allow for immediate and automatic evaluation of the facial function even before the facial nerve has been identified. Facial MEP was shown to be particularly useful in large tumors where identification of the proximal facial nerve occurs at an advanced stage of the surgery [1]. Further, wave amplitude of the facial MEP has shown a correlation with postoperative facial function according to the HB scores.

#### Cochlear nerve monitoring

Cochlear nerve (CN) monitoring and preservation is more difficult to achieve, as there are no stimulation methods that allow clear electrophysiological mapping. Brainstem auditory evoked response (BAER), with preservation of waves I and V, and cochlear compound action potentials (CAP) are useful intraoperative tools to preserve CN function in small-medium sized tumors [57, 117, 131] but not in larger tumors. This evidence belongs to a case series of patients undergoing VS surgery for small-medium sized tumors when hearing preservation was attempted [57, 117, 131]. Available data supporting IOM for hearing preservation in patients undergoing surgery for large VS are extracted from recent series of STR focusing on a “nerve-centered approach” [31, 158]. In this setting continuous BAER monitoring with defined alert criteria such as reduction of peak III amplitude of more than 50% has been used [31]. However, the delay from data averaging to obtaining a waveform and detecting a change in the BAER may prevent the surgeon from actually altering intraoperative strategy to have a positive impact on hearing.

Direct CN recordings may elicit larger amplitudes leading to a “real-time” CN assessment and may overcome the limits of the previous techniques [32, 154]. This is a more technically demanding type of monitoring due to the difficulties in placing the electrodes at the nerve root entry zone and in keeping and securing the probes in place during the intervention, especially in large VS. Due to the lack of comparative studies, no clear evidence has proved the superiority of direct nerve recordings over BAER. A few series [32, 131] reported hearing preservation outcome using this technique and results are not adjusted for the tumor size or surgical resection technique; therefore, no recommendation can be drawn for large VSs.

*The literature supports the routine use of IOM during* VS *surgery to preserve FN and CN function when preoperative hearing is present. Free-running electromyography and evoked compound muscle action potential mapping has become standard of care for facial nerve monitoring. (Level C)*

*When hearing preservation is attempted, the literature supports the use of BAER and/or direct CN monitoring. No evidence supports the superiority of direct CN action potentials over BAERs. (Level C)*

### Radiosurgery and radiation therapy

Patients with large VSs are usually not considered for upfront SRS, because of the need for surgical decompression in clinically symptomatic patients and the risk of further clinical deterioration during transient tumor expansion after SRS [70, 113]. Nevertheless, a few authors have assessed the feasibility and safety of primary SRS in asymptomatic large VS, reporting long-term tumor control up to 94% with good functional outcome and low complication rates [67, 94]. Lefranc et al. [94] reported the results of a large case series of a subgroup of 86 asymptomatic or minimally symptomatic patients with large VSs (defined as Koos Grade IV) treated by GKRS as first-line treatment. After a mean follow-up of 6.2 years, tumor control with no clinical deterioration was achieved in 90.7% and no brainstem or cranial nerve toxicity was observed. Huang et al. [67] reported similar results with a tumor control of 85.7% without further neurological deterioration after a median follow-up of 48 months. Van de Langenberg reported a series of GKRS in 33 patients with large VS (defined as a tumor > 6 cm^3^ and at least indenting the brainstem) [175]. Tumor control was achieved in 88% of cases, with clinical control (defined as no need for further treatment) in 79% of cases. Hearing preservation was achieved in 58% of cases and normal facial nerve function in 91%. Though the exact definition of “large” VSs varies between the series considered, several authors have shown that larger tumor volume (> 15 cm^3^), brainstem compression and/or displacement of the fourth ventricle resulted in failed disease control and tumor progression.

Fractionated stereotactic radiation therapy (FSRT) regimens include the use of conventional radiation therapy (e.g., 50.4–57.6 Gy in 1.8–2.0 Gy daily fractions, 5 times per week) or hypofractionated regimens (e.g., 5 Gy daily × 5; 3 Gy daily × 10; 4 Gy daily × 10; 6 Gy daily × 3). For the particular case of VSs, there are, in the current literature, 6 nonrandomized trials comparing single-fraction SRS versus FSRT [3, 4, 27, 28, 84, 106]. With regard to tumor control, 5-year rates between SRS (95–100%) and FSRT (91–100%) were similar [3, 4, 27, 28, 84, 106]. Facial nerve preservation rates were not statistically different at 5 years [167]. With regard to hearing preservation, 5-year rates ranged between 33 and 85% in the SRS group versus 44–86% in the FSRT group [3, 4, 27, 28, 84, 106]. The indications for fractionation remain, in our opinion, limited. Whether there is a cutoff volume where FSRT may be favored over SRS is currently unknown. In cases with contraindication to microsurgical resection, radiation therapy, either by SRS or by FSRT, can be an alternative [69, 94].

As previously described, over the last decade, many centers have integrated SRS and STR as part of a planned combined approach for large VSs with tumor control rates up to 96% and functional nerve preservation up to 93% and 80% for FN and cochlear nerve respectively [158] (Table 2). Nevertheless, there is no evidence regarding the timing of SRS for a tumor remnant after a NTR/STR to compare the superiority of a waiting policy with SRS as a salvage treatment vs. upfront SRS to the residual tumor, as part of a combined treatment paradigm. Troude et al. [165] reported on outcome after adjunct GKRS after subtotal resection of large VS. Of a historical cohort of 143 patients without GTR, 66 (46.2%) were allocated to a wait-and-scan policy and 77 (53.8%) to upfront GKRS. Of note, 27 patients (19.3%) presented with growing tumor remnants. The progression-free survival (LPFS) at 7 years was not statistically different between the two groups. The authors concluded that the low probability of long-term regrowth of small tumor remnants is an argument for a wait and re-scan policy with SRS as an option for salvage treatment. Some authors recommend SRS in the months following less than GTR when the size and location of the residual VS is suitable for optimal SRS dosimetry planning and treatment [31, 158].

#### Rescue surgery for large VS after SRS

Microsurgery after failed SRS maybe associated with worse cranial nerve outcomes and STR due to the poorer arachnoid planes surrounding the neurovascular structures following radiation [40, 68, 72, 92, 179]. However, others suggest no clear relationship between the use of SRS and the subsequent ease or difficulty of delayed microsurgery [91, 132]. In their recent literature review, Husseini et al. [68] reported a worsened FN function in rescue surgery in up to 83% of cases, difficult dissection between 43 and 100%, and rates of GTR between 0 and 89%. These results are in line with data published in the largest series [40, 68, 120, 141, 179], which agree that salvage surgery is associated with increased risk of nerve damage and greater difficulty in dissection. Caution should be exercised in interpreting this data as most series include a small number of cases, a heterogenous group of patients operated by surgeons of varying experiences [48].

Breshears et al. [15] reported a GTR in 7/10 cases and a postoperative HB 1 in 8/10 patients with sporadic VS previously treated with GKRS. These excellent results are not shared by others [48] who report poorer facial nerve outcomes after complete microsurgical removal of VSs following failed SRS as compared with those who had not undergone any prior intervention prior to primary resection. Despite the aforementioned criticisms, there is no consensus regarding the management of this subgroup of patients, and decisions depend on the surgeon’s own experience and surgical philosophy. Many surgeons agree that the goal of every VS surgery, especially in cases of failed previous surgical and/or radiosurgical treatment, should be GTR, as it is the only option that may provide a cure for the patient [15, 48]. On the other hand, other authors prefer a less-than-total resection followed by repeat SRS if there are adhesions to preserve cranial nerve function [72]. Microsurgery after SRS for VS is rarely indicated but the timing and techniques required can be challenging [90]. Repeat SRS is a feasible option when tumor growth is well documented in lieu of a rescue surgery for the aforementioned reasons [42], though literature review is sparse on this topic especially for large residual tumors. Obviously, this option should be weighed against a second combined management.

*According to the literature, upfront SRS is not recommended for large VS with mass effect. .(Level C)*

*There is no level I evidence to support the superiority of SRS as a salvage* versus *upfront treatment for tumor remnants after STR. Performing SRS during the 3–6 months after microsurgical resection has proved to be safe and effective. The factors influencing the timing of SRS include the shape and size of the residual lesion in addition to the recovery of any neural deficits that have occurred after surgery. (Level C)*

*Single-fraction SRS can be performed with low marginal radiation doses of 11–14 Gy, with high local tumor control, minimal risk of facial palsy and high hearing preservation rates in patients having good pretreatment hearing. (Level C)*

*Similar to previously untreated patients, patients with failed SRS should be counseled about the risk of functional nerve impairment associated with surgical treatment based on the aforementioned data reported in the literature and the specific strategy should be tailored according to the patient’s expectations. (Expert opinion)*

*The need for surgical resection after SRS should be reviewed with the neurosurgeon who performed the radiosurgery and should be delayed until sustained tumor growth is confirmed and after having ruled out pseudo-progression or radionecrosis. (Expert opinion)*

### Postoperative evaluation and quality of life after surgery

#### Facial palsy

Facial palsy can have a severe impact on patients’ QoL, affecting their emotional status and general and social health [29, 41, 87, 89, 118, 162, 168]. Unexpectedly, several studies have failed to detect a direct association between the severity of facial impairment and the level of psychological distress and/or level of social function. Consequently, the magnitude of this impact cannot be predicted by the severity of the FN deficit but rather by the patient’s perception of his/her own handicap [29, 41, 89]. Furthermore, the patient’s perception of his/her deficit has been shown to be far worse than the clinician’s estimation, which usually tends to underestimate the clinical outcome [41, 118] and that any level of FN impairment, defined as not normal (HB ≥ 2), can have a tremendous impact on QoL [89]. This discrepancy highlights the psychosocial component influencing self-evaluation and patients may report high levels of psychological distress and social dysfunction despite a “surgical satisfactory result” that are unlikely to improve with time [89, 118]. Obviously, the patient’s perception of his/her deficit depends on cultural factors and on personal expectations for surgical outcome. While in some countries, regaining employment has a positive influence on QoL possibly outweighing the negative effect of a FN palsy [168], in Western culture, a slight FN palsy can be perceived as a devastating disability [29, 41, 89, 118, 162]. Finally, the intermedius nerve (Wrisberg’s nerve) function needs to be assessed as persistent eye dryness can be a significant disability in some patients.

#### Unilateral hearing loss

Hearing preservation is most often ignored in the pursuit of a total excision for a large VS. However, even a unilateral hearing loss can have a serious impact on the daily life and communicative skills of these patients as loss of binaural hearing reduces the ability to localize sounds [59]. Several series on patients with unilateral hearing loss have reported reduced general quality of life (QoL) and slightly worse social function [80, 121, 170]. The current treatment for single-sided deafness is limited to observation, contralateral routing of signal hearing aids without auditory input into the involved ear [58], or implantation of bone-anchored hearing aids (BAHAs) [23, 80]. Through transcranial direct bone conduction to the contralateral cochlea, BAHAs eliminate the head shadow and enable patients to hear and communicate on the deaf side. These devices have been proven to restore some binaural hearing, thus improving the patients’ QoL [9]. While providing no benefit for sound localization, BAHAs improve speech discrimination in noisy surroundings, thereby diminishing the social, physical, and psychological stress. A systematic review on functional outcomes after cochlear implant (CIs) in patients with sporadic VSs reported a 30 to 56.4% improvement in mean speech discrimination score and an improvement in tinnitus [9]. Although the use of follow-up MRIs in patients with CIs is considered to be limited due to imaging artifacts [9], Carlson et al. [22] showed that under controlled conditions, 1.5-T MRI can successfully evaluate the ipsilateral skull base. Notwithstanding this limitation, select sporadic VS patients can be considered for CIs [9]. Proper counseling is required to ensure that patients are informed about long-term hearing prognosis at the time of diagnosis.

#### Tinnitus

Rates of reported postoperative tinnitus outcome are discordant and vary from 15 to 66% (resolved), 6 to 60% (improved), 10 to 90% (unchanged), and 6 to 50% (worsened) [10]. The pathophysiology of VS-associated tinnitus is still unclear. The main hypothesis suggests a peripheral origin in the acute phase due to nerve irritation, whereas in the chronic phase, a neuroplasticity occurs at the central level which is independent of the peripheral stimulus. It appears that anatomical cochlear nerve preservation, irrespective of functional hearing after surgery, is associated with a higher risk of developing postoperative tinnitus. Anatomical deafferentation in the setting of preoperative nonfunctional hearing or complete hearing loss has significantly reduced the risk of postoperative tinnitus [10] [164]; in fact, patients with better preoperative hearing had a bad prognosis with respect to postoperative tinnitus [81]. While cutting the cochlear nerve in cases where no hearing preservation is contemplated might reduce the risk of postoperative tinnitus [81], cochlear nerve deafferentation rarely relieves chronic tinnitus and will hamper a later CI.

Evaluation of the quality of life has gained importance in medicine in recent years and has resulted in a definite shift in the focus of physicians, from a clinical and technical standpoint toward a more patient-oriented treatment strategy that focuses on well-being. At present, the Penn Acoustic Neuroma Quality-of-Life (PANQOL) scale [85, 101, 151] is the only patient-reported QOL instrument validated for VS patients. This 26-item survey assesses patient-perceived QOL in seven domains as follows: hearing, balance, facial dysfunction, anxiety, energy, pain, and general health. Although this scale strongly correlates with the general Short Form-36 Health Survey (SF-36) in some domains, the PANQOL scale seems to reliably quantify discomfort associated with facial nerve deficit [151]. Nevertheless, the SF-36 is an established, cross-disease QOL tool used for validation of other questionnaires. The Facial Clinimetric Evaluation (FaCE) scale [78] that measures facial impairment and disability specifically evaluates the impact of facial palsy on QOL [38] and correlates significantly with the facial dysfunction domain of the PANQOL [101].

*Facial nerve function is of primary concern in large* VS *surgery. In the European context, this assumes great significance in the overall QoL. Therefore, patients should be counseled on the FN functional outcomes. The appropriate surgical strategy should be based on this evaluation and frank discussions with patient and family. (Expert opinion)*

*Hearing preservation for patients with preoperative useful hearing should also form part of the preoperative discussion pertaining to functional hearing outcome, QoL and postoperative hearing rehabilitation. All patients should be counseled about the non-negligible risk of persistence or new-onset tinnitus after surgery and also the limitations that exist with respect to proven treatment of this symptom. (Expert opinion)*

*Evaluation of the health-related quality of life represents a primary requirement in the management of patients with a* VS *and should be assessed before and after treatment. (Expert opinion)*

### Clinico-radiological follow-up

There is no uniform pattern across centers with respect to the frequency of surveillance after complete or incomplete resection of a large VS [31, 165].

Even in cases of GTR, postoperative MRI often shows a linear enhancement within the resection cavity which fades over time and sometimes can present the characteristics of a nodular enhancement due to the use of fibrin or tissue grafts [18, 20, 171]. High-resolution 3D T2-weighted imaging has not proven to be superior to a standard post-contrast T1-weighted imaging at identifying tumor recurrence or residual progression. Any development or progression of a nodular enhancement should be considered as a recurrence [11, 18, 20].

Although MRI has universally been adopted as the preferred imaging modality, the time sequence for follow-up images after SRS varies in the published literature based on institutional protocols. SRS series reporting long-term follow-up images agree on performing MRI 1 year after treatment but the intervals during the first year varied from 3 months to 6 months [105, 107, 114].

*In case of GTR, we recommend a follow-up MRI at 3–6 months and at 1 year from surgery and subsequently repeated every 2–5 years if no recurrence has been observed. (Level C)*

*In case of a less than total resection a more frequent surveillance is suggested with annual MRI scans. (Level C)*

*For any progressive or new nodular enhancing lesion suspicious of recurrence we recommend a* post-contrast T1-weighted MRI *after 6 months to document evolution of the lesion and the need for further treatment. (Level B)*

*In the case of STR (as a stand-alone approach or as a part of a combined approach), we recommend an MRI at 3–4 months to confirm that the residual tumor has a volume and anatomical relationship suitable for optimal radiosurgery planning and dosimetry. (Expert opinion)*

*After SRS, we recommend follow-up evaluation with MRI every 6 months for the first year and then annually or bi-annually based on clinical indications. (Level C)*

## Summary of recommendations

- *Tumor size (largest extrameatal diameter of the tumor and its volume) is to be used for reporting results. (Expert opinion)*
- *Tumor classification grades must be used when reporting the results of surgical series. (Expert opinion)*
- *Large* VS *are defined as tumors larger than 30 mm and giant tumors > 40 mm. (Expert opinion)*
- *The initial screening evaluation includes an audiometry for all patients with symptoms of vestibulocochlear nerve dysfunction and if this reveals an abnormality, patients should undergo a prompt screening MRI. (Level C)*
- *Classification or grading scales for pre- and postoperative hearing (AAO-HNS or GR*) *and facial nerve function (HB) need to be used when reporting the patient’s status and the outcome. (Expert opinion)*
- *The main goal of the management for VSs should focus on maintaining or improving QoL making every attempt at neurological function preservation in addition to optimal oncological control. (Expert opinion)*
- *There is insufficient evidence in literature to support the superiority of any surgical strategy (gross total resection* vs. *sub/near total with or without radiosurgery) (Level C)*
- *IOM should be routinely used during* VS *surgery to preserve facial and cochlear nerve function whenever possible. (Level C)*
- *Upfront SRS is not recommended for large* VS. *(Level C)*
- *If a planned combined approach (subtotal surgery followed by SRS) was the chosen management plan, SRS can be given 3–6 months after surgery based on the morphology of the tumor residue and recovery of any postoperative neurological deficits. (Level C)*
- *Single-fraction SRS can be performed with low marginal radiation doses of 11–14 Gy, with high local tumor control, minimal risk of facial palsy and high hearing preservation rates in patients having good pretreatment hearing. (Level C)*
- *Evaluation of the health-related quality of life including facial nerve function represents a primary requirement in the management of patients with a* VS *and should be assessed before and after treatment. (Expert opinion)*
- *Hearing preservation for patients with preoperative useful hearing should form part of the preoperative discussion pertaining to functional hearing outcome, QoL and postoperative hearing rehabilitation. (Expert opinion)*
- *All patients should be counseled about the non-negligible risk of persistence or new-onset tinnitus after surgery and also the limitations that exist with respect to proven treatment of this symptom. (Expert opinion)*
- *We recommend postoperative evaluation with* post-contrast T1-weighted imaging at 3–6 months with any *progressive or new nodular enhancement considered suspicious for recurrence. We recommend an MRI after 6 months in order to evaluate the evolution of the lesion and the need for further treatment. (Level B)*
- *In GTRs, we recommend follow-up with* post-contrast T1-weighted imaging *at 3–6 months and at 1 year from surgery and subsequently at intervals of 2–5 years if no recurrence is observed. (Level C)*
- *In STRs (as a stand-alone approach or as a part of a combined approach), we recommend an MRI at 3–4 months in order to ensure that the residual volume has a volume and anatomical relationship that is suitable for optimal radiosurgery planning and dosimetry. (Expert opinion)*
- *After SRS, we recommend follow-up evaluation with MRI every 6 months for the first year and then annually or bi-annually based on clinical indications. (Level C)*
